# Development of a checklist to detect errors in meta-analyses in systematic reviews of interventions: study protocol

**DOI:** 10.12688/f1000research.53034.1

**Published:** 2021-06-08

**Authors:** Raju Kanukula, Matthew Page, Kerry Dwan, Simon Turner, Elizabeth Loder, Evan Mayo-Wilson, Tianjing Li, Adya Misra, Steve McDonald, Andrew Forbes, Joanne McKenzie

**Affiliations:** 1 School of Public Health and Preventive Medicine, Monash University, Melbourne, VICTORIA, 3004, Australia; 2 Cochrane Methods Support Unit, Cochrane, London, SW1Y 4QX, UK; 3 Division of Headache, Department of Neurology, Brigham and Women's Hospital, Harvard Medical School, Boston, MA, 02115, USA; 4 The BMJ, BMA House, London, WC1H 9JP, UK; 5 Department of Epidemiology and Biostatistics, Indiana University School of Public Health-Bloomington, Bloomington, IN, 47405, USA; 6 Department of Ophthalmology, School of Medicine, University of Colorado Denver, Denver, CO, 80045, USA; 7 Department of Epidemiology, Johns Hopkins Bloomberg School of Public Health, Baltimore, MD, 21205, USA; 8 PeerJ, Inc, San Diego, CA, 92191, USA

**Keywords:** systematic review, meta-analysis, errors, checklist, reporting guideline, statistical issues, synthesis, pair-wise meta-analysis

## Abstract

**Background** *:* Systematic reviews underpin clinical practice and policies that guide healthcare decisions. A core component of many systematic reviews is meta-analysis, which is a statistical synthesis of results across studies. Errors in the conduct and interpretation of meta-analysis can lead to incorrect conclusions regarding the benefits and harms of interventions; and studies have shown that these errors are common. Enabling peer reviewers to better detect errors in meta-analysis through the use of a checklist provides an opportunity for these errors to be rectified before publication. To our knowledge, no such checklist exists.

**Objective** *:* To develop and evaluate a checklist to detect errors in pairwise meta-analyses in systematic reviews of interventions.

**Methods** *:* We will undertake a four-step process to develop the checklist. First, we will undertake a systematic review of studies that have evaluated errors in the conduct and interpretation of meta-analysis to generate a bank of items to consider for the checklist. Second, we will undertake a survey of systematic review methodologists and statisticians to seek their views on which items, of the bank of items generated in step 1, are most important to include in the checklist. Third, we will hold a virtual meeting to agree upon which items to include in the checklist. Fourth, before finalising the checklist, we will pilot with editors and peer reviewers of journals.

**Conclusion** *:* The developed checklist is intended to help journal editors and peer reviewers identify errors in the application and interpretation of meta-analyses in systematic reviews. Fewer errors in the conduct and improved interpretation will lead to more accurate review findings and conclusions to inform clinical practice.

## 1. Introduction

Systematic reviews (SRs) frequently underpin clinical practice guidelines and policies that guide healthcare decisions. A core component of many SRs is meta-analysis, a statistical technique used to synthesise study effect estimates from studies addressing similar questions, yielding a quantitative summary. ^1^ Extensions to meta-analysis (e.g. meta-regression, subgroup analysis) allow for investigation of factors that may explain variation of results across studies. These methods have the potential to provide valuable insights for healthcare decision-making; however, they are reliant on the methods being appropriately applied and interpreted.

Many errors can arise when conducting meta-analysis. For example, when meta-analysing continuous outcomes, calculations may be incorrect if standard errors are confused with standard deviations. When data are included from multi-arm trials, there is the risk that participants might be counted more than once when multiple comparisons from these trials are eligible for inclusion in the same meta-analysis. For example, from a three-arm trial of paroxetine, fluoxetine, and placebo, two comparisons would be eligible for a meta-analysis of ‘antidepressants versus placebo’ (i.e., ‘paroxetine versus placebo’ and ‘fluoxetine versus placebo’). When dealing with non-standard randomized trials – such as crossover trials, cluster-randomized trials, or split-body trials – there is a risk that variances of the effect estimates in the meta-analysis do not appropriately account for the correlation in observations induced by these designs. ^2^^–^^5^ Such errors can lead to studies receiving the incorrect weight in the meta-analysis with potential consequent impact on the combined estimate of intervention effect and its confidence interval, and other statistics, such as the estimated heterogeneity variance and measures of inconsistency. In some circumstances, these errors will lead to a different interpretation of the findings and review conclusions. ^6^

Statistical errors have been observed frequently in published SRs. For example, a study including 42 reviews from the Cochrane Cystic Fibrosis and Genetic Disorders Group found that nearly half of the SRs had at least one error (e.g., used standard error instead of standard deviation; calculated standard deviations incorrectly from the standard error given in the report; entered median instead of mean). ^7^ Another study in which the authors re-extracted the data from two randomly selected trials included in each of 27 meta-analyses, found errors in how the meta-analyst entered data for at least one of the two trials in 17 (63%) of the meta-analyses. ^6^ Furthermore, some published meta-analyses papers have been retracted because of errors in analyses and error in results and/or conclusions. ^8^^–^^10^

Research has shown that errors in the interpretation of statistical analyses in reviews are also common. For example, of 110 SRs indexed in MEDLINE® in 2014, 62 used the random-effects model, but 57 (92%) incorrectly interpreted the meta-analytic effect as the best estimate of a common intervention effect across studies, rather than as the average of the intervention effects across studies. In 42 of the 110 meta-analyses, a subgroup analysis was undertaken, but the findings were not interpreted with respect to a test for interaction in 29/42 (69%), and in 11/42 (26%), a claim of a subgroup difference was made based on a statistically significant effect in one group and not the other. ^11^ Furthermore, the potential for confounding by other factors as a possible explanation for observed subgroup effects, was not raised in any of the SRs. ^11^^,^^12^

Many strategies have been proposed to improve the conduct of meta-analysis (thus lessening the chance of errors occurring) and the interpretation of findings. These include, for example, textbooks, ^13^^–^^15^ training on meta-analysis methods, connection with support systems (e.g., Cochrane’s TaskExchange), and the inclusion of statisticians on review teams. However, even with these strategies, errors will still occur. A possible additional strategy is to enable peer reviewers to better detect possible errors in meta-analyses.

The peer-review process is regarded as a valuable approach for helping peer reviewers and journal editors to judge the quality, critically appraise and finally accept or reject the submitted manuscripts for publication. ^16^ Researchers have explored the impact of checklists to guide peer reviewers in assessing the completeness of reporting of submitted manuscripts, and have found some evidence that these are effective. ^17^^–^^19^ For example, training early career researchers to use the COBPeer tool (which is an online CONSORT-based peer-review tool assessing nine domains: the eight most important CONSORT domains and a switch in primary outcome(s)) helped them detect inadequate reporting in randomized trials compared to the usual review process. ^17^^,^^20^ To our knowledge, no such checklist has been developed to detect statistical errors in meta-analyses.

**Aim:** To develop and evaluate a checklist to detect conduct and interpretation errors in pairwise meta-analyses in systematic reviews of interventions.

## 2. Defining the concept of statistical errors

The notion of statistical conduct and interpretation errors is not simple. Brown and colleagues ^21^ defined errors to be “actions or conclusions that are demonstrably and unequivocally incorrect from a logical or epistemological point of view (e.g. … mathematical mistakes, statements not supported by the data, incorrect statistical procedures …)”. In this research, we will consider statistical errors to include those arising from underlying assumptions not being met, incorrect values used in the calculations, application of incorrect statistical methods, and misinterpretation of the results and statistical tests. We plan to initially group errors into categories (Section 3.2.4) and refine and revise these based on the types of errors identified through the systematic review (Section 3.2). Our focus will be on errors where it can be reasonably expected that a trained meta-analyst should have or could have known better, recognising that there is subjectivity in making this determination. ^21^

## 3. Methods

### 3.1 Contributors

A core team (RK, MJP, KD, SLT, EL, EMW, TL, AM, ABF, JEM) will lead the development of this checklist. The core team will conduct the systematic review, develop survey content and analyse survey responses, draft the checklist, coordinate piloting of the checklist, and decide the final content of the checklist. The core team consists of individuals with experience in meta-analysis methods and SR methodology, contributors of the Cochrane Handbook for Systematic Reviews of Interventions, and editors of medical journals who frequently publish SRs (BMJ, PLOS Medicine, Cochrane Database of Systematic Reviews, American Journal of Public Health, and Systematic Reviews).

### 3.2 Systematic review

We will conduct an SR of studies evaluating errors in the conduct and interpretation of pairwise meta-analysis, for the purpose of identifying types of errors, their prevalence, and to generate a bank of items to potentially be included in the checklist.

*3.2.1 Eligibility criteria*

Studies will need to meet the following eligibility criteria:

Inclusion criteria:

• Studies evaluating types of errors (and potentially their prevalence) in the conduct and interpretation of meta-analyses (and its extensions, for example, subgroup analysis, sensitivity analysis) in SRs of interventions (irrespective of included study design);
• Articles presenting a checklist or tool to evaluate the conduct of meta-analyses in SRs.

Exclusion criteria:

• Studies evaluating the methodological or reporting quality or risk of bias in SRs using a tool that does not specifically examine statistical errors (e.g. PRISMA, AMSTAR-2, ROBIS) **;**
• Studies or checklists evaluating errors in statistical analyses in primary study designs (e.g. randomized trials and observational studies).

*3.2.2 Search methods*

We will search MEDLINE, Embase and Scopus from inception to January 2021, without any publication type or language restrictions. The search strategies for MEDLINE and Embase combine subject heading terms and text words related to statistical errors in meta-analyses are presented in Appendix (see *Extended data*). ^22^ The search was iteratively developed and tested by an experienced information specialist (SM) using a set of 10 methods articles relevant to the topic. The Scopus search closely replicates the MEDLINE search with respect to included terms and word adjacency but is limited to the following subject areas in Scopus: medicine, nursing, dentistry and health professions.

We will also search abstracts of papers and posters presented at Cochrane Colloquia since 2011 (available at https://abstracts.cochrane.org/), including the Global Evidence Summit 2017. The search strategy will be determined by assessing the relative recall of terms from eligible studies identified from searches of MEDLINE, Embase and Scopus. We will screen the reference lists and conduct a cited reference search in Web of Science of included articles and review our personal collections of reports or studies related to statistical issues in meta-analyses. In addition, we will contact organisations that produce SRs (e.g., Cochrane, Campbell Collaboration, National Institute for Health and Care Excellence), and journals that frequently publish SRs to seek any in-house checklists they are willing to share.

*3.2.3 Selection of studies*

Two authors will screen independently all titles and abstracts according to the aforementioned eligibility criteria and retrieve the full text of any potentially relevant articles. The same authors will screen the full texts of retrieved articles. In case of any discrepancies, a senior author will adjudicate and finalise the inclusion or exclusion of any article(s).

*3.2.4 Data collection*

Once we finalise the studies to be included, two authors will collect data independently from each article using a standardised data collection form. For studies evaluating types of errors (and potentially their prevalence), we will collect the following information: corresponding author name, email address, year of publication, journal name, objective(s), focus of error investigation (e.g., multi-arm trials, cross-over trials, cluster randomized trials), type and prevalence of errors, and recommendations provided for conducting meta-analyses. For articles presenting a checklist or tool to evaluate conduct or reporting of meta-analyses in SRs, we will collect the following information: checklist/tool name, method of checklist/tool development, number of items included in the checklist/tool and scope of the checklist/tool. In addition, we will collect all the items and response options pertinent to meta-analysis and its interpretation identified in the tools/checklists, and these will be added to our item bank.

Once we have extracted data from all articles, we will review the items in the item bank and remove any duplicate or redundant items. We will then group items into broader categories. For example, those relating to data type (e.g., continuous, binary), rare outcomes (i.e., handling of zero events in one or both arms), design of included studies (e.g. cross-over, cluster, multi-arm, non-randomised [e.g. interrupted time series, cohort]), type of analysis (meta-analysis, subgroup analysis, meta-regression, sensitivity analysis, publication bias analysis) and issues of interpretation. We will use Microsoft Excel 2016 for data management.

### 3.3 Survey

We will send an invitation to all SR methodologists and statisticians (identified from the Cochrane Methods community, Campbell Methods Coordinating Group, Society for Research Synthesis Methodology and other SR methodologists and statisticians known to the core team members) and SR editors and statistical editors (identified from Cochrane Review Groups, and those supporting other journals that frequently publish SRs) to complete a survey to inform the development of the checklist. The survey will ask respondents to:

1) Provide their views on the most important items from the bank of items (generated from the systematic review) to include in the checklist. We will ask respondents to prioritise items that capture/identify the most common and consequential errors expected to occur in the conduct and interpretation of meta-analyses;
2) Provide their views on specific signals (or ‘red flags’) that might lead them to conduct a more thorough investigation of statistical errors in reviews (e.g., size of the effect for some studies, meta-analysis methods used, I-squared value etc.).

We will ask researchers to provide rationale for their responses and to suggest additional items not listed in the survey. We will calculate frequencies of each response option for each item and specific signals. For an item or specific signal to meet consensus for discussion, one of the response options for the item or signal will need to be selected by more than 70% of survey respondents; this threshold was selected according to Sumsion 1998 *et al*. ^23^

### 3.4 Virtual meeting

Following the survey, the core team will hold a virtual meeting to agree upon which items to include in the statistical errors’ checklist for editors/peer reviewers, which items might trigger further investigation (by a statistical reviewer or the authors) and discuss how best to word each item. Attendees will discuss all items exceeding the 70% threshold in the survey. We will also send the items rated as important by fewer than 70% to meeting attendees prior to the meeting, to provide them with the chance of “rescuing” items for discussion at the meeting. Following the meeting, the core team will draft the checklist and an accompanying guidance document (with examples for each item).

### 3.5 Piloting

In the first stage of piloting, two reviewers will read the draft checklist and guidance document before independently applying it to a random sample of 20 reviews; 10 from each of two previous methodological studies that collated systematic reviews. Specifically, the first methodological study includes 42 SRs of nutrition research (the ROBUST study) ^24^ that were published between January 2018 and June 2019, and the second includes 31 SRs of interventions for arthritis, depression or anxiety (the SIM study) that were published between January 2010 and January 2012. ^25^ The reviewers will record issues on whether the wording of items is ambiguous or difficult to interpret, and those items will be discussed by the core team and improved.

In the second stage of piloting, we will invite associate editors and peer reviewers of journals that frequently handle SR submissions to pilot the checklist and provide feedback on its usability. After collating the feedback received from the peer reviewers and editors, we will finalise the checklist and the accompanying guidance document.

### 3.6 Dissemination and knowledge translation strategy

We plan to publish the developed checklist and guidance document in an open-access journal. We will disseminate the checklist via presentations and workshops at relevant conferences and workshops focused on SR methodology, health technology assessment and evidence-based medicine (e.g., Cochrane Colloquia, Evidence Live, HTAi Annual Meeting, The Global Evidence Summit, G-I-N Conference), and via social media, and in a series of international webinars. We also plan to create a user-friendly, online version of the checklist and guidance document for use by journals that publish SRs and meta-analyses.

The developed checklist and related work will be published in open access journals. Associated datasets, data collection forms and analyses not included in any publication will be made publicly available via an online repository.

## 4. Conclusion

The developed checklist could help journal editors and peer reviewers identify errors in the conduct and interpretation of meta-analyses in SRs. Fewer errors and improved interpretation would lead to more accurate SR findings and conclusions to inform clinical practice.

**Study status:** We have completed searching and screening for sections 3.2.1, 3.2.2 and 3.2.3 for systematic review.

## Data availability

### Underlying data

No underlying data are associated with this article.

### Extended data

Bridges: Search strategy for systematic review of studies evaluating errors in the conduct and interpretation of pairwise meta-analysis. Monash University. DOI: https://doi.org/10.26180/14446293.v1. ^22^

This project contains the following extended data:

- Search strategy for systematic review of studies evaluating errors in the conduct and interpretation of pairwise meta-analysis

Data are available under the terms of the Creative Commons Attribution 4.0 International license (CC-BY 4.0).
