# Supplementary material for: Development of a checklist to detect errors in meta-analyses in systematic reviews of interventions: study protocol
Source: F1000Res. 2021 Jun 8;10:455. [Version 1] doi: 10.12688/f1000research.53034.1 (PMC8258702; doi:10.12688/f1000research.53034.1)
Supplement: Peer review history

# Peer review: Reviewer response for version 1

**DOI:** 10.5256/f1000research.56375.r87112

**Published:** 2021-07-05

**Authors:** Therese D. Pigott

**Affiliations:** 1 School of Public Health and College of Education and Human Development, Georgia State University, Atlanta, GA, USA

This work will provide an important contribution to the field, highlighting typical errors that can occur in pairwise meta-analyses of intervention effects. One question I have is the audience of the checklist. The study protocol is situated in meta-analysis for health and medical interventions. The search strategy for relevant systematic reviews for stage one of the process focuses on the health and medical literature, with only the Campbell Collaboration as a source for meta-analyses in the social sciences. If the intent of the checklist is to apply more broadly to social interventions rather than just health and medicine, then the search strategy needs to include a broader set of databases. There are relevant efforts in psychology for example (see Maasen et al. (2020 ^1^)) that could also inform the checklist. I understand if the authors wish to focus this effort only on health and medicine. 

 In the social sciences, similar issues to those that are outlined in the protocol occur. In addition, many meta-analyses include a large number of studies, and thus key issues center on dependent effect sizes within studies and interpretation of effect size models for heterogeneity. If the authors intend the checklist to have a broader application, then these are a few issues that should be considered in the development of the checklist.

Is the study design appropriate for the research question?

Yes

Is the rationale for, and objectives of, the study clearly described?

Yes

Are sufficient details of the methods provided to allow replication by others?

Yes

Are the datasets clearly presented in a useable and accessible format?

Yes

Reviewer Expertise:

meta-analysis methods

I confirm that I have read this submission and believe that I have an appropriate level of expertise to confirm that it is of an acceptable scientific standard.

## References

[1] : Reproducibility of individual effect sizes in meta-analyses in psychology. *PLoS One* .2020;15(5) : 10.1371/journal.pone.0233107 e0233107 10.1371/journal.pone.0233107 32459806PMC7252608

# Peer review: Reviewer response for version 1

**DOI:** 10.5256/f1000research.56375.r87114

**Published:** 2021-06-21

**Authors:** Ram Bajpai

**Affiliations:** 1 School of Medicine, Keele University, Staffordshire, UK

Authors have well prepared their systematic review protocol with clarity. I have following minor suggestions to improve the quality of this protocol.

 From inclusion criteria it is clear that authors will include SRs of interventions only. However, if possible, authors should elaborate on not including SR of proportions or SR of observational studies or SR of biomarkers etc. Each set of SRs will require a different type of statistical skills to perform meta-analysis. It also important to mention about whether only pharmacological intervention SRs or non- pharmacological interventions such as educational interventions etc. will also be included in this methodological systematic review.

 Did authors think that a statistician included in the author list will have any impact on error as it is possible that SRs with statistician as co-author may have less statistical errors? I think the authors have already thought about this point, however, appreciate authors reflection on this point and if they add few words in the protocol.

 If I am not wrong, the planned survey and consensus meeting will follow Delphi survey methodology. I am wondering that authors did not mention this word explicitly or they are following some other equally useful methodology.

 I believe that authors have well refined methodology to capture statistical errors. However, in many SRs, authors wrongly choose a meta-analysis model (i.e., fixed-effect vs. random-effect) to pool study estimates and possible other statistics right. How would authors capture such errors? Please elaborate if I have missed this point.

Is the study design appropriate for the research question?

Yes

Is the rationale for, and objectives of, the study clearly described?

Yes

Are sufficient details of the methods provided to allow replication by others?

Yes

Are the datasets clearly presented in a useable and accessible format?

Yes

Reviewer Expertise:

Meta-analysis, Statistical analysis

I confirm that I have read this submission and believe that I have an appropriate level of expertise to confirm that it is of an acceptable scientific standard.
